# The varied influence of ocular *Demodex* infestation on dry eye disease and meibomian gland dysfunction across different age groups

**DOI:** 10.1038/s41598-023-43674-x

**Published:** 2023-09-28

**Authors:** Won Jun Lee, Minjeong Kim, Seung Hyeun Lee, Yeoun Sook Chun, Kyoung Woo Kim

**Affiliations:** 1 Department of Ophthalmology, Chung-Ang University College of Medicine, Chung-Ang University Hospital, 102 Heukseok-ro, Dongjak-gu, Seoul, 06973 Republic of Korea; 2 Biomedical Research Institute, Chung-Ang University Hospital, Seoul, Republic of Korea

**Keywords:** Medical research, Eye diseases, Corneal diseases, Eye abnormalities, Eyelid diseases

## Abstract

This study aimed to investigate the impact of ocular demodicosis on dry eye disease (DED) and meibomian gland dysfunction (MGD) across different age populations: young (20 to < 40), middle-aged (40 to < 60), and elderly (≥ 60), based on the retrospective medical chart review. In each age subgroup, *Demodex* infestation and its count were correlated with clinical parameters of DED and MGD. Among the total of 351 subjects, 52.7% had ocular demodicosis, with a mean of 2.31 ± 1.39 mites per four eyelashes (0.58 per lash) in a unilateral eye. In the age subgroup 1 (age < 40; N = 44), subjects with *Demodex* had significantly higher meibum quality grades. In subgroup 2 (40 ≤ age < 60; N = 122), subjects with *Demodex* had higher ocular surface disease index scores and higher MG expressibility grades. However, in subgroup 3 (age ≥ 60; N = 185), demographics and all parameters did not differ according to *Demodex* infestation. Moreover, the number of mites did not correlate with MGD severity in any of the subgroups. In conclusion, age may act as a significant confounding factor in the relationship between ocular *Demodex* infestation and clinical features of DED and MGD, despite older patients aged 60 years and above being at a higher risk of *Demodex* infestation and experiencing more severe MGD.

## Introduction

*Demodex* mites are parasitic creatures measuring 150–350 μm in length that are commonly found in the eyelid area. While there are several *Demodex* species, *Demodex folliculorum* (*D. folliculorum*) and *Demodex brevis* (*D. brevis*) inhabit the human body^1^. The former resides in eyelash follicles, while the latter is found in meibomian and sebaceous glands of the eyelashes, particularly on the ocular surface^2^. The infestation of these mites has been linked to conditions such as anterior blepharitis, meibomian gland dysfunction (MGD), and even corneal abnormalities^3^.

*D. folliculorum* has been identified as a vector for bacteria, fungi, and viruses^4^, and may directly penetrate cell membrane to feed, potentially inducing allergies^5^. Moreover, *Demodex* infestation may cause superficial corneal neovascularization, phlyctenule-like lesions, corneal infiltration, and opacity^6–9^. A recent study, conducted by ocular surface disease experts agreed that itching is the most common symptom in *Demodex* blepharitis^10^.

The relationship between *Demodex* infestation and meibomian gland health remains debated, with studies suggesting both associations and non-associations. A study indicated that ocular *Demodex* infestation might contribute to ocular surface discomfort, inflammation, and meibomian gland dropout in MGD patients^5^. Conversely, a study found no significant association between the expressibility and drop-out of meibomian glands and *Demodex* infestation^3^. Additionally, *D. brevis,* which usually inhabits the meibomian glands, has been suggested as a possible cause of repeated chalazia and hordeola rather than MGD in the posterior eyelid^11^. A recent randomized clinical trial used lotilaner ophthalmic solution to treat *Demodex* blepharitis, using the cure of collarette as a primary end point, a clinical marker of *Demodex*-associated anterior blepharitis^12^.

Earlier studies have shown that the prevalence of ocular *Demodex* increases with age^13–15^, and one study reported a demodicosis frequency of 77% in individuals aged over 70 years, compared to 8% in those aged 25 years or younger^16^. Furthermore, it was observed that the higher prevalence of ocular *Demodex* in elderly patients (41–70 years) was associated with more severe MGD compared to younger patients (18–40 years)x^15^. However, the pathogenic role of *Demodex* in MGD is remains controversial, it is recognized that aging could be a significant confound both *Demodex* infestation and MGD and/or DED, which are age-related conditions.

To assess the effect of age, it is needed to divide chronological age into several periods more than two. Therefore, we divided the adult participants into traditional three age subgroups^17^: young (20 to < 40), middle (40 to < 60), and old age (≥ 60) to specifically assess the effect of age. In this study, we investigated the prevalence of ocular *Demodex* in a large number of patients (N = 351) and assessed age-related differences in the association between *Demodex* infestation and MGD profiles, as well as clinical DED parameters.

## Results

### Demographics, MMP-9, and clinical parameters of DED

A total of 351 subjects were included in the study. Among them, 166 subjects were categorized as the "No *Demodex* group," showing no *Demodex* infestation, while 185 subjects were part of the "*Demodex* group," with one or more *Demodex* mites (2.31 ± 1.39) per four epilated eyelashes in a single eye. The average number of mites per 4 eyelashes in a unilateral eye within the complete study population (N = 351) was recorded as 1.22 ± 1.53.

Comparing the demographic details, the *Demodex* group had an average age of 62.0 ± 12.5 years, significantly higher than the No *Demodex* group's average age of 55.4 ± 15.5 years (*p* < 0.001, Table 1). There was no significant difference in the percentage of females between the two groups (66.9% in the No *Demodex* group and 71.0% in the *Demodex* group, *p* = 0.493, Table 1). Regarding clinical parameters of DED, such as the ocular surface disease index (OSDI) score, tear matrix metalloproteinase (MMP)-9 grades, tear osmolarity (Tosm), Sjogren's International Collaborative Clinical Alliance (SICCA) ocular staining score (OSS), tear break-up time (BUT), and tear secretion measured by Schirmer I without anesthesia, no significant differences were observed between the two groups (Table 1). However, the MGD profile grades, including MG expressibility and meibum quality, were worse in the *Demodex* group (1.63 ± 0.58 and 1.83 ± 0.63, respectively) than in the No *Demodex* group (1.48 ± 0.64 and 1.55 ± 0.81, respectively; *p* = 0.018 and *p* < 0.001, respectively, Table 1).

**Table 1:** Differences in demographics and clinical parameters of dry eye disease (DED) between subjects with and without ocular *Demodex* infestation in total subjects.

Variables	Group		*p* Value
	Without *Demodex*	With *Demodex*	
Total No. of subjects	166 (47.3%)	185 (52.7%)	–
Number of *Demodex* mites (per 4 eyelashes in a unilateral eye)	0	2.31 $\pm$ 1.39	–
Demographics			
Age (yrs)^a^	55.4 $\pm$ 15.5	62.0 $\pm$ 12.5	$<$ 0.001*
Male/female (% female)^b^	55/111 (66.9%)	55/130 (71.0%)	0.493
Clinical parameters			
OSDI (score)^a^	39.2 $\pm$ 20.3	40.4 $\pm$ 20.7	0.689
Tear MMP-9 (Gr)^a^	1.97 $\pm$ 1.13	2.1 $\pm$ 1.1	0.270
Tear osmolarity (mOsm/L)^a^	307.5 $\pm$ 46.7	307.8 $\pm$ 22.6	0.545
Corneal erosions score^a^	0.98 $\pm$ 1.78	1.01 $\pm$ 1.71	0.497
SICCA OSS^a^	1.11 $\pm$ 1.83	1.08 $\pm$ 1.72	0.938
Tear BUT (s)^a^	5.47 $\pm$ 2.04	6.02 $\pm$ 2.42	0.153
Schirmer I without anesthesia (mm)^a^	11.93 $\pm$ 9.07	11.20 $\pm$ 8.20	0.752
MG expressibility (Gr)^a^	1.48 $\pm$ 0.64	1.63 $\pm$ 0.58	0.018*
Meibum quality (Gr)^a^	1.55 $\pm$ 0.81	1.83 $\pm$ 0.63	$<$ 0.001*

*OSDI* ocular surface disease index, *MMP-9* matrix metalloproteinase 9, *OSS* ocular staining score, *BUT* break-up time, *MG* meibomian gland, *Gr* grade.

^a^Mann–Whitney U test, ^b^Chi-square test, *p < 0.05.

Significant values are in bold.

Furthermore, the *Demodex* count exhibited positive correlations with age (*r*_*s*_ = 0.240, *p* < 0.001), MG expressibility (*r*_*s*_ = 0.144, *p* = 0.008), and meibum quality grades (*r*_*s*_ = 0.180, *p* = 0.001, Table 2). However, no significant correlations were found between the number of mites and other clinical parameters of DED.

**Table 2:** Correlation between *Demodex* count and clinical parameters of dry eye disease (DED) in a population of total subjects.

Count of *Demodex* (per 4 eyelashes in a unilateral eye) vs	*r*_*s*_	*p* Value
Age (yrs)	0.240	$<$ 0.001*
OSDI (score)	0.048	0.506
Tear MMP-9 (Gr)	0.073	0.198
Tear osmolarity (mOsm/L)	− 0.057	0.572
Corneal erosions score	0.032	0.602
SICCA OSS	0.027	0.747
Tear BUT (s)	0.033	0.653
Schirmer I without anesthesia (mm)	− 0.091	0.103
MG expressibility (Gr)	0.144	0.008*
Meibum quality (Gr)	0.180	0.001*

*OSDI* ocular surface disease index, *MMP-9* matrix metalloproteinase 9, *OSS* ocular staining score, *BUT* break-up time, *MG*, meibomian gland, *Gr* grade.

Spearman’s rank correlation test, *p < 0.05.

Significant values are in bold.

### Prevalence of ocular *Demodex* in different age subgroups

Given the parallel increase in MGD severity and *Demodex* count with aging (as seen in Table 2), the total subjects were divided into three age-based subgroups: subgroup 1 (age < 40), subgroup 2 (40 ≤ age < 60), and subgroup 3 (age ≥ 60). The prevalence of ocular *Demodex* in these subgroups was 34.1%, 42.6%, and 63.8%, respectively (Table 3). Subgroup 3 showed a significantly higher prevalence of *Demodex* compared to subgroups 1 and 2 (*p* < 0.001 and *p* < 0.001, respectively, Table 3). The mean number of *Demodex* mites was also highest in subgroup 3 (1.54 ± 1.63) and significantly lower in subgroup 1 (0.59 ± 0.95, *p* < 0.001) and subgroup 2 (0.99 ± 1.47, *p* = 0.002, Table 3).

**Table 3:** Prevalence and counts of the ocular *Demodex* mites by age.

Variables	Age subgroup		
	*Subgroup 1* (age $<$ 40) (N = 44)	*Subgroup 2* (40 $\le$ age $<$ 60) (N = 122)	*Subgroup 3* (age $\ge$ 60) (N = 185)
Prevalence of ocular *Demodex*	34.1%	42.6%	63.8%
Comparison of prevalence^a^	*p* Value		
*versus subgroup 1*	–	0.323	$<$ 0.001*
*versus subgroup 2*	–	–	$<$ 0.001*
Number of mites (per 4 eyelashes in a unilateral eye)	0.59 $\pm$ 0.95	0.99 $\pm$ 1.47	1.54 $\pm$ 1.63
Comparison of counts^b^	*P* Value		
*versus subgroup 1*	–	0.559	$<$ 0.001*
*versus subgroup 2*	–	–	0.002*

^a^Chi-square test, ^b^Kruskal–Wallis test with Dunn’s post-hoc analysis, *p < 0.05.

Significant values are in bold.

### Differences in demographic and clinical parameters of DED with and without *Demodex* infestation across different age subgroups

In subjects under 40 years old (subgroup 1), demographics and clinical parameters of DED, except for meibum quality grade, did not differ significantly based on the presence of *Demodex* infestation. The severity grade of meibum quality was significantly higher (2.00 ± 0.71) in subjects with *Demodex* compared to those without *Demodex* (0.96 ± 0.86, *p* = 0.001, Table 4).

**Table 4:** Differences in demographics and clinical parameters of dry eye disease (DED) between subjects with and without ocular *Demodex* infestation in subgroup 1.

Variables	*Subgroup 1* (age $<$ 40) (N = 44)		*p* Value
	No *Demodex*	*Demodex*	
No. of subjects	29	15	–
Number of *Demodex* mites (per 4 eyelashes in a unilateral eye)	0	1.73 $\pm$ 0.80	–
Demographics			
Age (yrs)^a^	29.8 $\pm$ 6.6	33.9 $\pm$ 4.1	0.060
Male/female (% female)^b^	18/11 (37.9%)	5/10 (66.7%)	0.070
Clinical parameters			
OSDI (score)^a^	44.7 $\pm$ 19.4	32.2 $\pm$ 17.0	0.187
Tear MMP-9 (Gr)^a^	1.96 $\pm$ 1.08	2.00 $\pm$ 1.18	1.000
Tear osmolarity (mOsm/L)^a^	317.6 $\pm$ 23.4	329.0 $\pm$ 25.1	0.400
Corneal erosions score^a^	1.60 $\pm$ 2.37	1.25 $\pm$ 1.60	0.769
SICCA OSS^a^	2.38 $\pm$ 3.29	4.50 $\pm$ 1.73	0.200
Tear BUT (s)^a^	5.69 $\pm$ 1.84	7.10 $\pm$ 2.47	0.139
Schirmer I without anesthesia (mm)^a^	18.52 $\pm$ 11.44	17.09 $\pm$ 4.44	0.978
MG expressibility (Gr)^a^	1.44 $\pm$ 0.58	1.67 $\pm$ 0.62	0.294
Meibum quality (Gr)^a^	0.96 $\pm$ 0.86	2.00 $\pm$ 0.71	0.001*

*OSDI* ocular surface disease index, *MMP-9* matrix metalloproteinase 9, *OSS* ocular staining score, *BUT* break-up time, *MG* meibomian gland, *Gr* grade.

^a^Mann–Whitney U test, ^b^Chi-square test, *p < 0.05.

Significant values are in bold.

For subjects aged 40–59 years old (subgroup 2), the OSDI score and MG expressibility grade in those with *Demodex* infestation were significantly higher compared to those without *Demodex* (45.2 ± 17.7 vs. 37.7 ± 20.2, *p* = 0.042; 1.57 ± 0.57 vs. 1.33 ± 0.62, *p* = 0.021, respectively, Table 5).

**Table 5:** Differences in demographics and clinical parameters of dry eye disease (DED) between subjects with and without ocular *Demodex* infestation in subgroup 2.

Variables	*Subgroup 2* (40 $\le$ age $<$ 60) (N = 122)		*p* Value
	No *Demodex*	*Demodex*	
No. of subjects	70	52	–
Number of *Demodex* mites (per 4 eyelashes in a unilateral eye)	0	2.33 $\pm$ 1.40	–
Demographics			
Age (yrs)^a^	52.4 $\pm$ 5.5	53.9 $\pm$ 4.9	0.145
Male/female (% female)^b^	17/53 (75.7%)	21/31 (59.6%)	0.058
Clinical parameters			
OSDI (score)^a^	37.7 $\pm$ 20.2	45.2 $\pm$ 17.7	0.042*
Tear MMP-9 (Gr)^a^	1.94 $\pm$ 1.23	2.00 $\pm$ 1.03	0.814
Tear osmolarity (mOsm/L)^a^	301.1 $\pm$ 64.15	312.1 $\pm$ 19.4	0.896
Corneal erosions score^a^	0.93 $\pm$ 1.73	1.03 $\pm$ 1.84	0.963
SICCA OSS^a^	1.23 $\pm$ 1.69	1.12 $\pm$ 1.88	0.585
Tear BUT (s)^a^	5.47 $\pm$ 1.80	5.76 $\pm$ 1.96	0.580
Schirmer I without anesthesia (mm)^a^	10.32 $\pm$ 6.81	11.71 $\pm$ 8.99	0.672
MG expressibility (Gr)^a^	1.33 $\pm$ 0.62	1.57 $\pm$ 0.57	0.021*
Meibum quality (Gr)^a^	1.44 $\pm$ 0.69	1.63 $\pm$ 0.66	0.103

*OSDI* ocular surface disease index, *MMP-9* matrix metalloproteinase 9, *OSS* ocular staining score, *BUT* break-up time, *MG* meibomian gland, *Gr* grade.

^a^Mann–Whitney U test, ^b^Chi-square test, *p < 0.05.

Significant values are in bold.

However, in subjects aged 60 or above, all demographics and clinical parameters of DED did not show significant differences based on Demodex infestation (Table 6).

**Table 6:** Differences in demographics and clinical parameters of dry eye disease (DED) between subjects with and without ocular *Demodex* infestation in subgroup 3.

Variables	*Subgroup 3* (age $\ge$ 60) (N = 185)		*p* Value
	**No *Demodex***	***Demodex***	
No. of subjects	67	118	–
Number of *Demodex* mites (per 4 eyelashes in a unilateral eye)	0	2.37 $\pm$ 1.44	–
Demographics			
Age (yrs)^a^	69.7 $\pm$ 6.9	69.2 $\pm$ 7.0	0.625
Male/female (% female)^b^	20/47 (70.1%)	29/89 (75.4%)	0.435
Clinical parameters			
OSDI (score)^a^	39.3 $\pm$ 21.0	38.8 $\pm$ 22.2	0.729
Tear MMP-9 (Gr)^a^	2.00 $\pm$ 1.05	2.18 $\pm$ 1.10	0.316
Tear osmolarity (mOsm/L)^a^	311.3 $\pm$ 20.3	302.2 $\pm$ 22.5	0.242
Corneal erosions score^a^	0.80 $\pm$ 1.56	0.97 $\pm$ 1.68	0.274
SICCA OSS^a^	0.57 $\pm$ 1.26	0.68 $\pm$ 1.11	0.469
Tear BUT (s)^a^	5.39 $\pm$ 2.37	5.95 $\pm$ 2.56	0.290
Schirmer I without anesthesia (mm)^a^	11.15 $\pm$ 9.25	10.38 $\pm$ 7.89	0.961
MG expressibility (Gr)^a^	1.65 $\pm$ 0.64	1.64 $\pm$ 0.58	0.936
Meibum quality (Gr)^a^	1.86 $\pm$ 0.77	1.91 $\pm$ 0.58	0.772

*OSDI* ocular surface disease index, *MMP-9* matrix metalloproteinase 9, *OSS* ocular staining score, *BUT* break-up time, *MG* meibomian gland, *Gr* grade.

^a^Mann–Whitney U test, ^b^Chi-square test.

### Association of *Demodex* count with MGD severity grades and OSDI Scores

The count of *Demodex* mites did not exhibit a significant correlation with the severity grades of MG expressibility and meibum quality, and OSDI scores in the entire population of subjects with ocular *Demodex* infestation. Furthermore, no correlation was observed with either grade and score when data was categorized by age (Table 7).

**Table 7:** Correlation between *Demodex* count with severity grade of meibomian gland dysfunction (MGD) and ocular surface disease index (OSDI) scores in subjects with *Demodex* infestation across different age subgroups.

Count of *Demodex* (per 4 eyelashes in a unilateral eye) vs	With *Demodex*					
	*Subgroup 1* (age $<$ 40) (N = 15)		*Subgroup 2* (40 $\le$ age $<$ 60) (N = 52)		*Subgroup 3* (age $\ge$ 60) (N = 118)	
	*r*_*s*_	*p* Value	*r*_*s*_	*p* Value	*r*_*s*_	*p* Value
MG expressibility (Gr)	0.222	0.389	0.159	0.260	0.067	0.474
Meibum quality (Gr)	0.442	0.097	− 0.027	0.850	0.011	0.903
OSDI (score)	− 0.311	0.495	0.128	0.470	− 0.005	0.971

*MG* meibomian gland, *Gr* grade. Spearman’s rank correlation test.

## Discussion

Several prior studies have established correlations between ocular *Demodex* infestation and conditions such as anterior blepharitis, MGD, chalazia, and keratoconjunctivitis^11,14,18^. The prevalence of ocular demodicosis and the count of infested *Demodex* mites have been shown to increase with age^13,15^, and DED is widespread, affecting 5–50% of the global population^19^. A significant portion of DED cases is attributed to MGD^20^. Given the well-established influence of aging on DED and MGD development^21^, we explored whether the higher prevalence of *Demodex* infestation in older individuals significantly contributes to DED and MGD in elderly populations. Surprisingly, our findings indicate that ocular demodicosis in those over 60 years old may not be linked to the severity of MGD and DED, despite the elevated prevalence of ocular *Demodex* and mite counts in comparison to younger individuals.

Conversely, ocular demodicosis seemed to influence meibum quality negatively in subjects below 40 years old. As indicated by Liang et al.^11^, *D. brevis*, typically residing in meibomian glands, was more common among younger patients with recurring chalazia and hordeola. Furthermore, MGD was detected in 90% of patients with D. brevis under 35 years, and it exhibited a significant correlation with keratitis^22^. Although this study observed worsened meibum quality in individuals under 40 years old with Demodex infestation, the subjective discomfort, as measured by OSDI scores, did not show significant differences between those with and without ocular demodicosis. Previous research also noted a connection between Demodex infestation and meibum quality in young patients, without a corresponding difference in OSDI scores, consistent with our findings^15^.

Gao et al. first investigated lipidomic changes associated with *Demodex* occurrence and they discovered that the levels of (O-acyl)-ω-hydroxy fatty acids (OAHFAs) were elevated in young adults aged 18–40 with ocular demodicosis, employing a liquid chromatography-mass spectrometry system^23^. This finding is intriguing, as OAHFA is recognized for its role in stabilizing the tear film through the creation of an interface between the non-polar lipid sublayer and the aqueous phase layer^24,25^. While this is a hypothetical explanation, it is suggested that the meibomian gland may adaptively adjust the levels of OAHFAs to alleviate ocular surface discomfort caused by *Demodex* mites^23^. Similarly, the potential contribution of ocular demodicosis to ocular discomfort has yielded inconsistent and controversial results across various studies. For instance, symptom profiles did not significantly differ between individuals with and without *Demodex* when evaluated using the Dry Eye Questionnaire 5 and OSDI^26^.

In this investigation, ocular demodicosis was linked to a more pronounced level of MG expressibility and elevated OSDI scores among middle-aged individuals aged over 40 but under 60 years. Ayyildiz et al. observed a similar pattern, demonstrating a significant correlation between OSDI scores and *Demodex* infestation in individuals who were initially diagnosed with DED and fell within the age range of 40–68 years^27^. This aligns with findings from the Hirado-Takushima Study conducted in Japan, which reported a substantial increase in the prevalence of MGD during the fifth decade of life, surging to 21.6% compared to the 5.6% rate in the fourth decade.According to the Hirado-Takushima Study in Japan^28^, the prevalence of MGD in the 5th decade of age abruptly elevated to 21.6% compared to that in the 4th decade, which was 5.6%.

Among elderly individuals (aged ≥ 60 years), no correlations between ocular demodicosis and any parameters were observed in this study. This contrasts with the findings of a study by Sun et al.^15^, who categorized their study population using a cutoff of 40 years and discovered that elderly subjects (aged 41–70 years) with *Demodex* infestation exhibited more severe OSDI scores, lower fluorescein tear film BUT, increased corneal fluorescein staining, higher MG dropout, MG orifice plugging, abnormal lid margins, and elevated MG expression compared to those without *Demodex*^15^. Additionally, they proposed that the greater number of mites and possibly the prolonged duration of ocular demodicosis might contribute to the heightened susceptibility of the ocular surface's deterioration caused by *Demodex* in their study^15^. In fact, while the prevalence of ocular demodicosis was 51.5% in their study involving 202 Chinese subjects^15^, akin to the prevalence of 52.7% in our study involving the Korean population (Table 1), the average number of *Demodex* mites per patient was notably higher at 5.36 ± 6.20 per 12 eyelashes (0.45 mites per an epilated eyelash)^15^, compared to 0.30 mites per lash in our study. Furthermore, the number of mites in the Chinese elderly population with ocular demodicosis, defined as individuals over 40 years, was even more substantial at 10.64 ± 7.50 per 12 lashes (0.89 mites/lash)^15^, surpassing the 2.37 ± 1.44 per 4 lashes (0.59 mites/lash) in our study for individuals with ocular demodicosis aged 60 years or older. Cheng et al. reported 4.2 ± 3.9 mites per 8 eyelashes (0.53 mites/lash) in a *Demodex*-positive population with 68.9% prevalence of ocular demodicosis^26^, similar to the 2.31 ± 1.39 mites per 4 eyelashes (0.58 mites/lash) in our study, and observed no differences in OSDI scores between subjects with and without *Demodex* infestation^26^. In contrast, the Caucasian population demonstrated a mean of only 0.2 mites per epilated lash, and neither OSDI scores nor the comorbid MGD proportion were elevated in conjunction with ocular *Demodex* infestation^3^. In summary, we suggest that the varying mite counts among elderly subjects from different races and countries could play a substantial role in influencing changes in DED and/or MGD-related parameters.

Interestingly, within the elderly population, no correlation was observed between *Demodex* count and OSDI score, as evident from the data presented in Table 7 of this study. Furthermore, among subjects with *Demodex* infestation, OSDI scores did not exhibit significant differences across the three age subgroups (details not presented). Notably, typical symptoms associated with *Demodex* blepharitis, such as itching and redness^10^, did not find comprehensive representation in the OSDI questionnaire. The OSDI primarily addresses matters like photophobia, pain, sensations of foreign bodies, and blurring, which might not fully encapsulate the true symptoms of ocular demodicosis in the elderly subgroup.

We propose an alternative explanation: the chronicity of ocular demodicosis, DED, and/or MGD might trigger compensatory stress-relieving signals both physically and psychologically, similar to what is observed in Sjogren’s syndrome, a representative chronic condition^29^. This mechanism could involve the desensitization or inhibition of polymodal nociceptors due to prolonged or repeated activation^30^ caused by long-lasting but not severe inflammatory stimuli induced by demodicosis^14^. This hypothesis is supported by the predominance of *Demodex*-induced keratitis in the young population^22^ which aligns with our proposed mechanism. Additionally, in younger patients, the proportion of *D. brevis* is higher than that of *D. folliculorum,* which is associated with repeated hordeola and chalazia^11^. Definite signs of demodicosis in young individuals, such as keratitis, hordeolum, and chalazion, may be easily linked to complaints of subjective ocular surface discomfort, even with a smaller number of *Demodex* than the elderly.

Our study's findings are subject to limitations arising from its retrospective design. Furthermore, our investigation did not uncover morphological and structural changes of meibomian glands using meibography. Additionally, we opted not to distinguish between D. folliculorum and D. brevis throughout our analyses. Although prior research has indicated that these two species cause blepharitis in distinct locations, a study highlighted that chronic blepharitis linked to D. folliculorum could impact ocular surface function, leading to a range of ocular signs, symptoms, and contributing to MGD-related dry eye and a higher incidence of chalazion^31^. Consequently, we chose to combine both species for correlational analysis with clinical parameters. However, the exact proportion of *D. folliculorum* and *D. brevis* within our cohort remains unknown. Additionally, the retrospective inclusion of hospital-based patients for assessing subjective symptoms might introduce selection bias. Therefore, it is essential to exercise cautious interpretation before concluding that ocular demodicosis, particularly in the elderly population, does not significantly prompt ocular surface discomfort and DED and/or MGD.

For the tear BUT estimation, we utilized the standard strip method instead of employing the quantified liquid dye drop technique. Although it was known that the standard strip method exhibited high test sensitivity and specificity in discriminating between aqueous tear deficiency DED and MGD compared to normal conditions, and the standard strip method might not differ significantly from the liquid dye instillation method^32^, the tear BUT estimation using fluorescein dye with a fixed volume and concentration, or through non-invasive BUT estimation, would be a better method to establish the relationship between tear film instability and ocular demodicosis.

To our current knowledge, no prior study has examined MGD and DED parameters in relation to ocular *Demodex* infestation across three distinct age subgroups: the young-aged, middle-aged, and old-aged, categorized with age cutoffs of 40 and 60 years. In conclusion, ocular demodicosis did not exhibit an association with the severity of MGD and DED in patients aged over 60 years. Consequently, it is essential to approach the interpretation of *Demodex* mite presence in the eyelids of elderly individuals with MGD or DED as a potential pathogenic factor with caution. Similarly, careful consideration is necessary when contemplating *Demodex* irradiation treatment decisions.

In summary, it is imperative to acknowledge that age can manifest as a noteworthy confounding factor within the context of the correlation between ocular *Demodex* infestation and the clinical attributes of both DED and MGD. This phenomenon persists despite the higher susceptibility of individuals aged 60 years and older to *Demodex* infestation, as well as their propensity to encounter more pronounced manifestations of MGD.

## Methods

This study was a retrospective cross-sectional comparative cohort study. The whole process properly followed the tenets of the Declaration of Helsinki. The research protocol received approval from the Chung-Ang University Hospital Institutional Review Board (IRB), and considering the retrospective nature of the study design, the requirement for informed consent was waived by the IRB (Approval No. 2011-006-19339).

### Subjects

We identified consecutive 351 individuals who were referred to our institute’s dry eye disease (DED) clinic due to ocular surface discomfort and subsequently underwent examinations for clinical parameters related to DED and meibomian gland dysfunction (MGD), as well as diagnostic testing for ocular demodicosis between September 2019 and December 2020. Evaluation for ocular demodicosis was routinely conducted for all patients at our DED clinic who experienced ocular surface discomfort. We excluded subjects who had a systemic immunologic disease, including Sjogren’s syndrome and allergic disease. Additionally, individuals with pterygium were excluded. We also excluded those who were under systemic immune-related treatments or were using topical administration of anti-inflammatory eye drops or anti-glaucomatous eye drops. Furthermore, participants who had worn contact lenses within the previous three months were excluded, as were those who had undergone ocular surgery within the previous six months.

All patients were classified into three distinct age subgroups according to their age: subgroup 1 (age < 40), subgroup 2 (40 ≤ age < 60), and subgroup 3 (age ≥ 60).

### Study design

Our study design, based on a retrospective medical chart review, was as follows:

1. Compare demographics and clinical parameters of DED and MGD between subjects with and without ocular demodicosis among all subjects.
2. Correlate age and clinical parameters of DED and MGD with the number of *Demodex* mites among all subjects.
3. Compare the prevalence of ocular demodicosis and the number of *Demodex* mites between subjects in each age subgroup.
4. Compare of demographics and clinical parameters of DED and MGD between subjects with and without ocular demodicosis in each age subgroup.
5. Correlate the number of *Demodex* mites with MGD severity grades only in subjects with *Demodex* infestation in each age subgroup.

### Examination for ocular *Demodex* infestation

In our investigation, we thoroughly examined a total of eight eyelashes (four lashes per eye) for each patient. This examination was conducted under a slit-lamp microscope. From both the upper and lower lids, we extracted two eyelashes—specifically, one from each half of each lid. Following the method proposed by Gao et al.^33^, we prioritized lashes exhibiting cylindrical dandruff, also referred to as “sleevs”. These individual lashes were placed onto a slide and covered with a coverslip. To facilitate examination, a 20 μL droplet of saline was gently applied to the coverslip's edge using a pipette. Subsequently, the specimens were meticulously assessed under an optical microscope set at a magnification of 400×. During this examination, *Demodex* mite counts were meticulously documented for each eye separately.

For analyses, the eye with the greater count of *Demodex* mites was chosen to avoid duplicate reflection of OSDI scores within a subject. In instances where the mite count was the same in both eyes, one eye was chosen at random.

### DED parameters and MGD severity profiles

As part of our evaluation of DED parameters, we systematically assessed the following factors in the specified sequence: tear osmolarity (Tosm), levels of tear matrix metalloproteinase (MMP)-9, tear secretion through Schirmer I without anesthesia, tear break-up time (BUT), Sjogren’s International Collaborative Clinical Alliance (SICCA) ocular staining score (OSS), corneal erosion scores according to the National Eye Institute/Industry (NEI) grading scale, expressibility of the meibomian glands (MG), and quality of the secreted meibum. Furthermore, to gauge the subjective ocular symptoms of DED and their impact on vision-related function, we employed the ocular surface disease index (OSDI) questionnaire^34^. The assessment for ocular *Demodex* infestation was conducted after the comprehensive evaluation of DED and MGD.

Tosm was assessed using an I-PEN osmometer (I-MED Pharma Inc., Montreal, QC, Canada). To perform the measurement, a single-use disposable sensor (SUS) was inserted into the I-PEN device. Patients were instructed to open their eyes, and the tip of the SUS was gently placed at a 30°–45° angle, directly onto the palpebral conjunctiva on the inner side of the retracted lower eyelid, ensuring good contact with the palpebral conjunctiva's surface. After maintaining this position for a few seconds, the handheld osmolarity system emitted an audible beep and presented the osmolarity reading in milliosmoles per liter on its LCD screen^35^.

The tear MMP-9 test was conducted using InflammaDry (Quidel, San Diego, CA, USA), and we followed the guidelines outlined in the product documentation^36^. To collect tear fluid, a sterile sample collector was gently applied to various areas along the lower palpebral conjunctiva. Subsequently, the collected tear fluid was placed into the immunoassay test cassette. After activating the solution with buffer for 20 s, we verified the intensity of the red line within a designated readout window. In order to assess the diagnostic significance of the MMP-9 assay, we analyzed the results employing a 5-scale grading method. This grading system involved evaluating the depth of the red readout band observed during on-site inspection of MMP-9. Grades were assigned as follows: grade 0 indicating a negative result, grade 1 indicating a trace result, grade 2 indicating a weak positive result, grade 3 indicating a positive result, and grade 4 indicating a strong positive result. These gradings were determined based on a standardized system of photographs that had been established previously^37^.

Tear secretion was assessed through the Schirmer I test, a procedure that entailed positioning a Schirmer standard strip (Eagle Vision, Memphis, TN, USA) at the outer 1/3 point of the lower conjunctival fornix. The strip was left in place for 5 min, during which time tear fluid was absorbed, and no analgesic eyedrops were utilized.

The tear break-up time (BUT) measurement was conducted at least 15 min subsequent to the Schirmer I test, in adherence with established protocols^38^. The process involved placing a droplet of normal saline on a strip paper coated with fluorescein dye (Haag-Streit International, Koniz, Switzerland), followed by gently shaking off the excess. The prepared strip was then delicately positioned on the lower lid margin to facilitate staining of the tear film. The precise moment at which the initial tear film disruption was observed under a cobalt blue filter after the last blink was considered the BUT. This measurement was replicated three times using a stopwatch, and the resultant average value was utilized for analysis.

The ocular staining score was determined by observing each eye through a slit-lamp equipped with a yellow filter following the administration of fluorescein^39^. We obtained the SICCA score^40^ and NEI score^41^ according to established criteria. To assess MGD, we employed two distinct methods: examining MG expressibility of five glands within the central upper lid and evaluating the quality of secreted meibum. The grading for MG expressibility of meibum from five glands ranged from 0 to 3, where 0 indicated all glands were expressible, 1 indicated 3–4 glands were expressible, 2 indicated 1–2 glands were expressible, and 3 indicated no glands were expressible. Additionally, meibum quality was graded from 0 to 3, with each score corresponding to clear, cloudy, cloudy particulate fluid, and toothpaste-like consistency based on previously defined criteria^42^. A consistent assessment of all clinical parameters related to dry eye disease (DED) was carried out by a single experienced researcher (K.W.K).

The participants were instructed to fill out the OSDI questionnaire, which aimed to evaluate their personal perceptions of ocular symptoms associated with DED and its impact on visual function. The survey was intended to encompass a one-week timeframe leading up to the survey date and encompassed three distinct subscales: ocular symptoms, vision-related daily function, and environmental triggers. A total of twelve questions were presented to the patients, with responses rated on a scale ranging from 0 to 4, where 0 denoted "none" and 4 denoted "always." To determine the OSDI score, the sum of the scores was multiplied by 25 and then divided by the number of questions that were accurately answered^43^.

### Statistical analysis

For statistical analysis, we used SPSS software version 20.0 (SPSS, Inc., Chicago, IL, USA) and Prism software v.8.4.3 (GraphPad, La Jolla, CA, USA). Initially, if the data exhibited a normal distribution with standard deviation, a parametric test was utilized for analysis; otherwise, a non-parametric test was applied. To compare continuous variables between groups, we employed either the parametric Student’s *t*-test or the non-parametric Mann–Whitney U test. In order to compare categorical data between groups, the Chi-square test was employed. The correlation between continuous variables was assessed using the non-parametric Spearman’s rank correlation test. The datasets are presented as averages and standard deviations (±). Statistical significance was considered at a level of *p* < 0.05.

## Data Availability

The datasets generated and analyzed in the current study are available from the corresponding author on reasonable request.
